# Effect of echocardiographic imaging view and methods on left ventricular wall-thickness measurements in normal cats and cats with hypertrophic cardiomyopathy

**DOI:** 10.1093/jvimsj/aalag062

**Published:** 2026-04-08

**Authors:** Giovanni Grosso, Karsten Eckhard Schober

**Affiliations:** Department of Veterinary Sciences, University of Pisa, Pisa, Italy; Department of Veterinary Clinical Sciences, College of Veterinary Medicine, The Ohio State University, Columbus, OH, United States; Department of Veterinary Clinical Sciences, College of Veterinary Medicine, The Ohio State University, Columbus, OH, United States

**Keywords:** 2D transthoracic echocardiography, feline cardiology, HCM, interventricular septum, left ventricular free wall

## Abstract

**Background:**

Echocardiographic measurements of left ventricular free wall (LVFW) and interventricular septum (IVS) thickness are essential for diagnosing hypertrophic cardiomyopathy (HCM).

**Hypothesis/Objectives:**

To evaluate agreement between different imaging views and modes for assessing LV wall thickness in cats. We hypothesized that there is clinically relevant bias between methods, and that results cannot be used interchangeably.

**Animals:**

Two-hundred eighty cats; 140 controls and 140 with subclinical HCM.

**Methods:**

Retrospective, single-center, and cross-sectional study. End-diastolic IVS and LVFW thickness was evaluated by 2-dimensional (2D) and time-motion mode (M-mode) echocardiography, from right parasternal long-axis (RPLax) and right parasternal short-axis (RPSax) views: method-1 (M1), RPLax using 2D; M2, RPLax using M-mode; M3, RPSax using 2D; and M4, RPSax using M-mode. Using 2D images, the thickest portion of the LV wall was measured. Methods were compared using repeated measurement ANOVA on ranks and the Bland–Altman method with bias and 95% limits of agreement (95% LOA).

**Results:**

In controls, IVS thickness was not different among the 4 methods (*P* = .67; bias 0.04-0.10 mm, 95% LOA −1.09 to 1.28 mm); whereas LVFW thickness was (*P* < .001) using M-mode compared to 2D (bias 0.22-0.30 mm; 95% LOA −0.81 to 1.25 mm). In HCM cats, thickness of the IVS (bias −1.07 to −1.13 mm; 95% LOA −3.05 to 0.84 mm) and LVFW (bias 0.22 to −.27 mm; 95% LOA −2.09 to 2.03 mm) was different among methods (*P* < .001).

**Conclusions and clinical importance:**

Two-dimensional and M-mode echocardiographic methods in the assessment of LV wall thickness cannot be used interchangeably, particularly in cats with HCM.

## Introduction

Two-dimensional (2D) transthoracic echocardiography is the clinical noninvasive gold standard test for diagnosing hypertrophic cardiomyopathy (HCM) in cats. According to the current consensus statement of the American College of Veterinary Internal Medicine (ACVIM) on feline cardiomyopathies,^1^ the echocardiographic assessment of left ventricular (LV) wall thickness is pivotal in the detection of HCM in cats. Both the thickness of the interventricular septum (IVS) and the LV free wall (LVFW) are routinely evaluated during echocardiographic screening for myocardial disease in cats.^1^

The assessment of LV wall thickness in cats relies on 2 different echocardiographic imaging techniques: 2D and M-mode.^1–5^ In order to obtain a comprehensive evaluation of LV wall dimensions, the assessment of the IVS and the LVFW from both the right parasternal long-axis (RPLax) and right parasternal short-axis (RPSax) views is necessary. The current ACVIM guidelines on feline cardiomyopathies^1^ have not recommended a preferred echocardiographic view or technique to assess LV wall thickness. Thus, it remains unknown which of the different methods (2D vs M-mode and RPLax vs RPSax) is to be used in the measurement of LV wall thickness in cats, and if using different methods and views lead to the same conclusions. A consensus on the best echocardiographic method to determine LV wall thickness in cats is lacking, and to the authors’ knowledge, prospective studies focusing on this topic have not been published.

Therefore, the primary objective of this study was to evaluate bias and agreement between different echocardiographic imaging views (RPLax vs RPSax) and techniques (2D vs M-mode) in the assessment of LV wall thickness in apparently healthy cats and in cats with HCM. Our main hypothesis was that there is clinically relevant bias with wide limits of agreement (LOA) between the 2 recording methods and 2 imaging views and that results using different methods for echocardiographic wall-thickness evaluation in cats cannot be used interchangeably. A secondary objective was to evaluate differences of LV wall-thickness measurements between the IVS and the LVFW. We hypothesized that wall-thickness measurements of the IVS are larger than those of the LVFW.

## Material and methods

This was a retrospective, single-center, and observational study. Medical records of the Veterinary Medical Center at the Ohio State University, Columbus, OH, from November 2013 until August 2023 were reevaluated. Cats that had undergone transthoracic echocardiography with a diagnosis of “normal cardiovascular findings” composed the control group. Cats with the diagnosis of subclinical “hypertrophic cardiomyopathy” composed the HCM group. No owner consent was needed considering the retrospective design of the study only using clinical and echocardiographic data.

### Study cohorts

Cats in the control group were deemed to be healthy based on history, clinical findings, and physical examination. They were primarily imaged at the request of breeders for screening, due to the presence of a soft heart murmur, or as part of a preanesthetic exam. In control cats, visual inspection of cardiac structures including the papillary muscles was the first step to echocardiographically define normality. If in doubt, a maximum end-diastolic LV wall thickness of < 6 mm,^1^^,^^6^ using 2D images and both RPLax and RPSax views were required for supportive evidence. If the heart subjectively appeared normal but the cat was very large (>9 kg) an arbitrary wall-thickness threshold slightly above 6 mm (≤6.2 mm) was permitted due to the known association between body weight and wall thickness in cats.^5^ In cats with HCM, focal, segmental, or global thickening of the IVS or LVFW ≥ 6 mm or abrupt changes of wall thickness of more than 50% within one segment that was < 6 mm were considered diagnostic for HCM.^1^^,^^2^^,^^6^^,^^7^ Exclusion criteria were evidence of dehydration based on history and clinical examination, hyperthyroidism (diagnosis made based on total thyroxine analysis if deemed necessary at the discretion of the attending clinician), and any other known endocrinopathy, previous or current systemic hypertension (systolic blood pressure > 160 mmHg), vomiting and diarrhea, treatment with pimobendan and diuretics, and any other condition capable of leading to abnormally large LV wall thickness.

### Echocardiography

Medical records were reviewed, eligible cats identified, and all echocardiograms remeasured by 1 investigator (G.G.). Blinding the observers regarding group allocation was not possible as the group (control [normal LV wall thickness], HCM [thick LV walls]) and the method (2D, M-mode, and imaging view) were readily visible from the images to be evaluated. Myocardial segments were measured in triplicate and reported as the average value. Echocardiographic studies were performed by a board-certified cardiologist or resident in training supervised by a cardiologist. All echocardiograms and measurements were subsequently reviewed (within 3 months) by the principal investigator (K.E.S.) before data analysis. Transthoracic echocardiographic studies were performed using commercially available ultrasound systems (Vivid 7 Vantage and Vivid E95 with XDclear and with EchoPAC software version BT06, GE Medical Systems, Waukesha, WI, USA) and phased-array sector transducers with nominal frequencies of 7 and 10 MHz, depending on animal size and image quality. Prior sedation with butorphanol tartrate (0.15-0.25 mg/kg intramuscularly or intravenously; IVX Animal Health Inc., Miami, FL, USA) was permitted and not considered a violation of the inclusion criteria. Other sedatives were not used.

Standard echocardiographic images of the RPLax and RPSax views were acquired as previously described in dogs^8^ and cats.^4^ Gentle back and forth motion of the stored image loops using the trackball of the echocardiographic machine or the computer mouse of the EchoPac workstation assured the best identification of insertion points of false tendons and right ventricular structures in the measurement field (eg, chordae tendineae and papillary muscles) which were avoided.^9^

**Figure 1:**
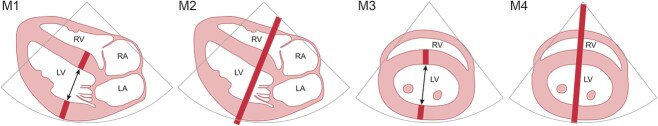
End-diastolic thickness of the IVS and LVFW were evaluated by 2D and M-mode echocardiography from both RPSax and RPLax views: M1 (method 1), assessment of IVS and LVFW thickness by 2D from the RPLax 4-chamber view; M2 (method 2), assessment of IVS and LVFW thickness by M-mode from the RPLax 4-chamber view; M3 (method 3), assessment of IVS and LVFW thickness by 2D from the RPSax view; and M4 (method 4), assessment of IVS and LVFW thickness by M-mode from the RPSax view. Abbreviations: 2D = 2-dimensional; IVS = interventricular septum; LA = left atrium; LV = left ventricle; LVFW = left ventricular free wall; RA = right atrium; RPLax = right parasternal long-axis; RPSax = right parasternal short-axis; RV = right ventricle. The bolded red lines indicate the location of left ventricular wall measurements.

End-diastolic thickness of the IVS and LVFW was evaluated by both 2D and M-mode in each cat from both RPSax and RPLax views according to reported techniques (Figure 1). Method 1 (M1) included the assessment of the thickness of the IVS and LVFW by 2D from the RPLax view (Figure 1); method 2 (M2) included the assessment of the thickness of the IVS and LVFW by M-mode from the RPLax view; method 3 (M3) included the assessment of the thickness of the IVS and LVFW by 2D from the RPSax view; and method 4 (M4) included the assessment of the thickness of the IVS and LVFW by M-mode from the RPSax view. If not previously recorded, anatomical M-mode for M2 and M4 was used to reconstruct the LV M-mode images by directing the M-mode cursor at the chordae tendineae level carefully avoiding the inclusion of the tips of the mitral valve leaflets and the bodies of the papillary muscles.^10^

As recommended in the ACVIM consensus statement,^1^ 2D measurements of the IVS (M1 and M3) were made using the leading edge-to-trailing edge technique and avoiding insertion points of false tendons, while 2D measurements of the LVFW were made using the leading edge-to-leading edge method (thus excluding the pericardium). When assessing the LV wall thickness by M-mode, 2D-guidance was used, and the IVS and LVFW were measured using the leading edge-to-leading edge technique.^4^ Using the latter, the LV endocardial layer (for IVS) and pericardium (for LVFW) are excluded. In cats with HCM, when assessing LV wall thickness by 2D (M1 and M3), the thickest portion of any segment of the IVS and LVFW was measured.^1^

### Evaluation of intra- and interobserver measurement variability

Reliability of echocardiographic measurements was evaluated.^11^ Intraobserver and interobserver measurement variability was calculated both as an absolute difference and the relative percentage difference with the coefficient of variation (CV) according to the following formula: CV (%) = (absolute mean difference of the measurements/average of the measurements) × 100.

For intraobserver measurement variability, the same investigator (G.G.) performed all measurements of wall-thickness variables from an arbitrary sample size of 10 randomly selected cats, of which 5 were controls and 5 had HCM, twice separated by approximately 24 h, in random order and in a blind fashion.

Interobserver measurement variability was determined by 2 investigators (G.G. and K.E.S.) performing all wall-thickness measurements from the same 10 randomly selected cats. Investigators were blinded to each other’s and previous measurements. Images or frames to measure were selected at the discretion of the investigator (G.G). The degree of reliability was classified as previously reported using the relative percentage difference: CV < 5% (excellent); CV 5%-15% (good); and CV > 15% (poor).^11^^,^^12^

### Statistical analysis

Using a freely available online sample size calculator (ClinCalc.com/stats/samplesize.aspx), assuming a group mean of 4-5 mm end-diastolic LV wall thickness for the control group and 6-7 mm for the HCM group and considering a > 1 mm difference between methods clinically relevant, with a SD of 1-2 mm (recently unpublished pilot data) and with an α of 5%, and a statistical power of 80%, approximately 126 cats in each group would be needed. Considering outliers and missing data, we aimed at a group size between 130 and 150 for control cats and cats with HCM. Statistical analysis was performed with commercially available software (Statistical Analysis Software, version 9.4, SAS Institute, Inc, Cary, NC and GraphPad Prism, version 8, GraphPad Software, Inc, San Diego, CA). All echocardiographic data were tabulated, visually inspected using dot plots, tested for normality using the Shapiro–Wilk test, and tested for outliers using Tukey’s method. Normally distributed data are reported as mean and SD, while non-normally distributed data are reported as median and minimum to maximum. Differences among the 4 echocardiographic methods (M1, M2, M3, and M4) for control cats and cats with HCM were compared using repeated measurement ANOVA on ranks. If a difference was identified, Tukey’s multiple comparison post-hoc test was used. Heteroscedasticity of residual plots to identify possibly increased (or decreased) differences between methods with increased (or decreased) magnitude of values was evaluated using the Breusch–Pagan test. The Bland–Altman (B-A) method with determination of bias and 95% limits of agreement (95% LOA) to graphically illustrate our findings. The value of *P* < .05 was used to determine statistical significance. Differences between control and HCM groups were evaluated with the *t*-test or Mann–Whitney rank-sum test, depending on the distribution of data. Differences between IVS and LVFW thickness within a particular method group (M1, or M2, or M3, or M4) were identified using a paired *t*-test or Wilcoxon signed-rank test.

## Results

**Figure 2:**
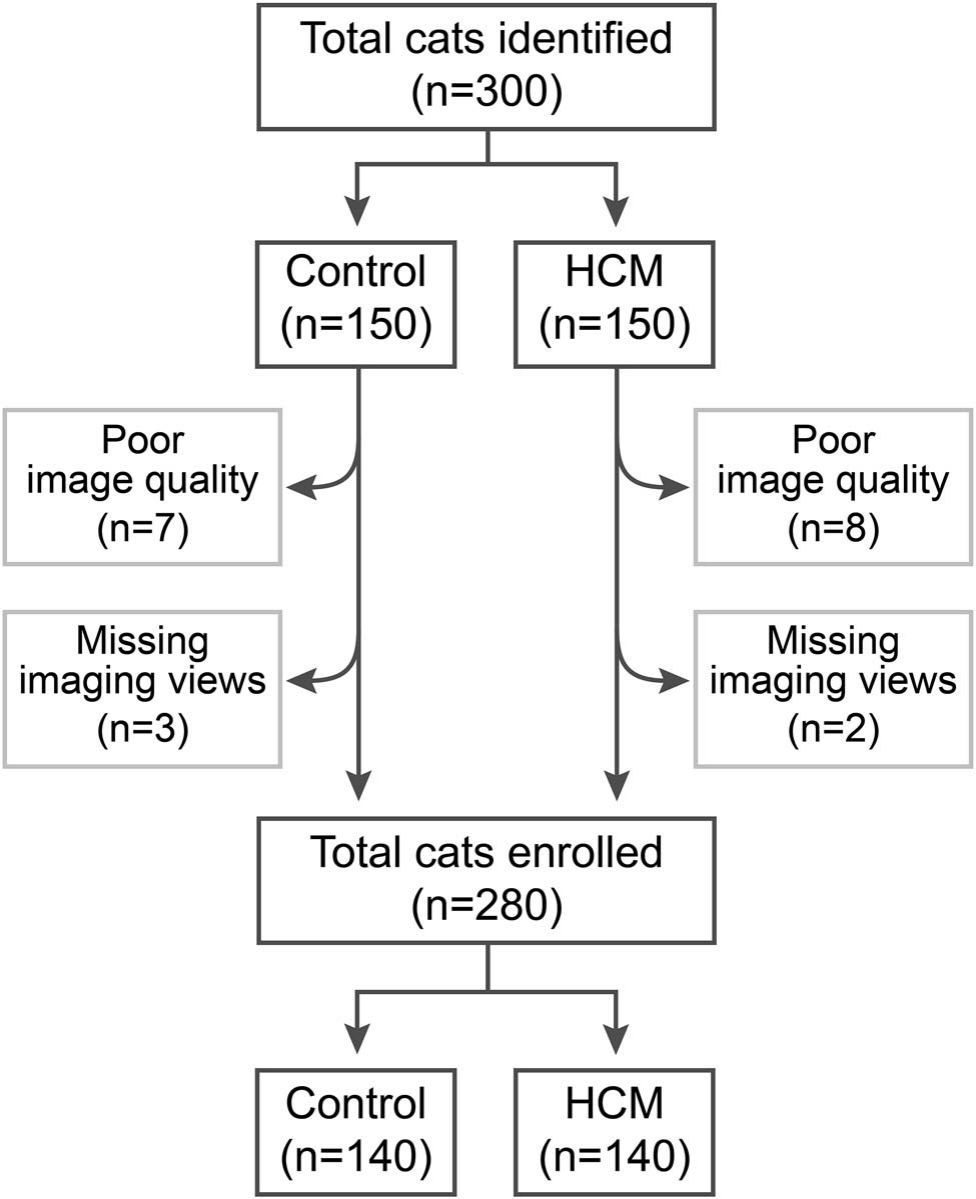
Flow chart demonstrating selection of study cats. Cats enrolled were imaged between November 2013 and August 2023, and the search terms “normal cardiovascular findings” and “hypertrophic cardiomyopathy” were used for case identification.

After initial review of medical records, a total of 300 cats were identified of which 20 (10 cats in each group) did not meet the inclusion criteria and thus were excluded. Therefore, data of 280 cats (140 in the control group and 140 cats in the HCM group) were studied (Figure 2). Data on sex, age, body weight, and breed are reported in Table 1.

**Table 1:** Summary of data on BW, sex, age, and breed in 140 control cats and140 cats with HCM.

	Control	HCM	*P*
**BW (kg)**	4.85 ± 1.53 (1.0-10.9)	4.96 ± 1.30 (2.5-7.8)	.45
**Sex (f/m)**	73/67	52/88	<.001
**Age (yrs)**	5.52 ± 4.79 (0.5-18.0)	10.0 ± 4.76 (0.7-20.2)	<.001
**Breed**	DSH (*n* = 56) Maine Coon (*n* = 37) Bengal (*n* = 17) DMH (*n* = 9) DLH (*n* = 6) Persian (*n* = 4) Sphynx (*n* = 4) Abyssinian (*n* = 2) One each Turkish Angora, ASH, Siamese, Turkish Van, Siberian	DSH (*n* = 78) DMH (*n* = 19) DLH (*n* = 16) Maine Coon (*n* = 7) Sphynx (*n* = 7) Himalayan (*n* = 3) ASH (*n* = 3) Ragdoll (*n* = 2) Bengal (*n* = 2) One each British SH, Scottish fold, Cornish Rex	n.d.
**Sedation^a^**	28	22	n.d.

a Butorphanol tartrate (0.15-0.25 mg/kg IM or IV).

Abbreviations: ASH = American shorthair; BW = body weight; DLH = domestic longhair; DMH = domestic medium hair; DSH = domestic shorthair; HCM = hypertrophic cardiomyopathy; n.d. = not determined.

Data reported as mean ± SD (minimum–maximum).

**Figure 3:**
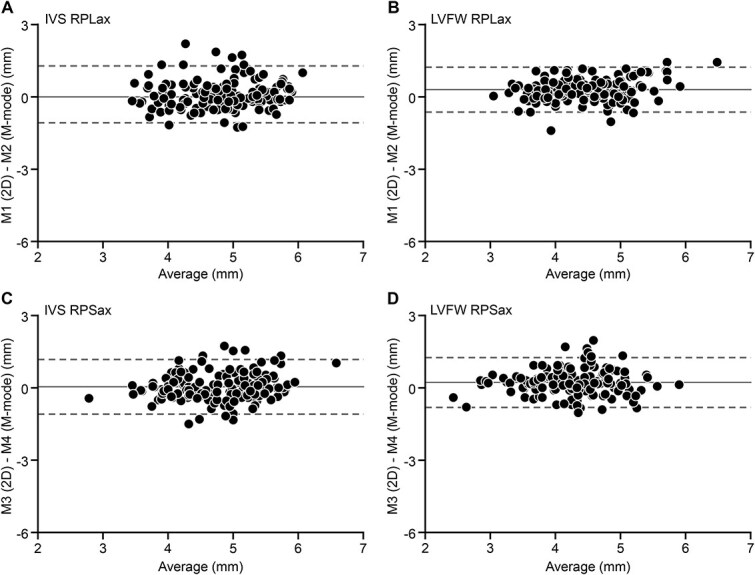
Bland–Altman plots illustrating difference (Y-axis) vs average (X-axis) of LV wall-thickness measurements using 4 echocardiographic methods in control cats. Individual data points, bias between methods (solid lines), and 95% LOA between methods (dashed lines) are displayed. (A) M1 vs M2 for the IVS. Bias 0.10 mm, SD of the bias 0.60 mm, 95% LOA −1.08 to 1.28 mm. (B) M1 vs M2 for the LVFW. Bias 0.30 mm, SD 0.47 mm, 95% LOA −.63 to 1.22 mm. (C) M3 vs M4 for the IVS. Bias 0.04 mm, SD 0.58 mm, 95% LOA −1.09 to 1.17 mm. (D) M3 vs M4 for the LVFW. Bias 0.22 mm, SD 0.53 mm, 95% LOA −0.81 to 1.25 mm. M, method; M1, right parasternal long-axis (RPLax) view, 2D measurements; M2, right parasternal long-axis (RPLax) view, M-mode measurements; M3, RPSax view, 2D measurements; M4, RPSax view, M-mode measurements. Abbreviations: 2D = 2-dimensional; IVS = interventricular septum; LOA = limits of agreement; LV = left ventricular; LVFW = left ventricular free wall.

In Table 2, echocardiographic data on thickness of the IVS and the LVFW using the 4 different methods (M1, M2, M3, and M4) in control cats and cats with HCM are reported. In control cats, thickness measurements of the IVS were not different among the 4 methods used (*P* = .67) with a fixed bias and a 95% LOA between 0.04 and 0.10 mm and −1.09 and 1.28 mm, respectively (Figure 3). In contrast, thickness measurements of the LVFW demonstrated differences (*P* < .001) between M1 and M3, M2 and M3, and M3 and M4 with fixed bias of 0.04-0.30 mm and 95% LOA between −1.09 and 1.28 mm in control cats (Figure 3). In general, the IVS was consistently thicker than LVFW (*P* < .001) for M1, M2, M3, and M4 in normal cats.

**Table 2:** Echocardiographic data on the thickness of the IVS and LVFW determined by 4 different methods (M1, M2, M3, and M4) in control cats and cats with HCM.

	RPLax view				RPSax view			
	2D (M1)		M-mode (M2)		2D (M3)		M-mode (M4)	
	IVSd (mm)	LVWFd (mm)	IVSd (mm)	LVWFd (mm)	IVSd (mm)	LVWFd (mm)	IVSd (mm)	LVWFd (mm)
**Control**	4.78 ± 0.68 (2.87-6.03)	4.33 ± 0.62^a^ (3.03-5.77)	4.88 ± 0.73 (3.30-6.57)	4.63 ± 0.73^b^ (3.07-7.20)	4.75 ± 0.67 (3.0-6.07)	4.15 ± 0.66^a,b,c^ (2.62-5.83)	4.79 ± 0.75 (2.57-7.10)	4.37 ± 0.68^c^ (2.23-5.97)
**HCM**	7.39 ± 1.22^a,b^ (4.3-10.5)	6.49 ± 1.33^e,f,g^ (3.37-9.77)	6.26 ± 1.23^c^ (2.43-9.50)	6.22 ± 1.38^e^ (3.20-10.07)	7.17 ± 1.17^a,c,d^ (4.30-10.77)	5.97 ± 1.38^e,f^ (2.70-10.30)	6.10 ± 1.31^b,d^ (3.17-9.50)	6.11 ± 1.51^g^ (1.37-12.07)

Data reported as mean ± SD (minimum–maximum).

Within a line (control and HCM), pairs sharing the same superscript letter are significantly (*P* < .05) different from each other.

In control cats, thickness of the IVS was not different among the 4 methods (*P* = .67) but thickness of the LVFW was (*P* < .001). There were differences between M1 and M3 (^a^, *P* = .02), M2 and M3 (^b^, *P* = .03), and M3 and M4 (^c^, *P* = .04) for the LVFW. In cats with HCM, thickness of the IVS was different among the 4 methods (*P* < .001). There were differences between M1 and M3 (^a^, *P* < .001), M1 and M4 (^b^, *P* < .001), M2 and M3 (^c^, *P* < .001), and M3 and M4 for the IVS (^d^, *P* < .001). In addition, thickness of the LVFW was also different among the 4 methods (*P* < .001). There were differences between M1 and M2 (^e^, *P* < .001), M1 and M3 (^f^, *P* < .001), and M1 and M4 (^g^, *P* = .01) for the LVFW.

Abbreviations: 2D, 2-dimensional ultrasound; HCM, hypertrophic cardiomyopathy; IVS, interventricular septum; LVFW, left ventricular free wall; M, method; M-mode, time-motion ultrasound; RPLax, right parasternal long-axis view; RPSax, right parasternal short-axis view.

**Figure 4:**
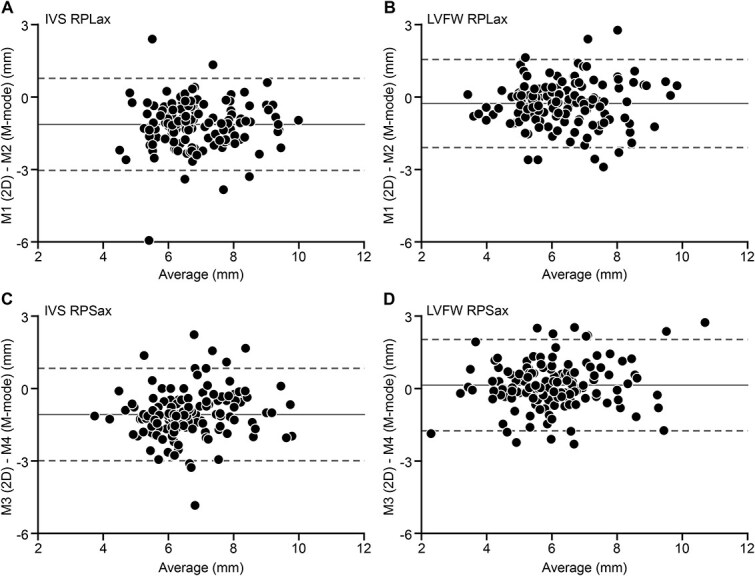
Bland–Altman plots illustrating difference (Y-axis) vs average (X-axis) of LV wall-thickness measurements using 4 echocardiographic methods in cats with hypertrophic cardiomyopathy. Individual data points, bias between methods (solid lines), and 95% LOA between methods (dashed lines) are displayed. (A) M1 vs M2 for the IVS. Bias −1.13 mm, SD 0.97 mm, 95% LOA −3.05 to 0.78 mm. (B) M1 vs M2 for the LVFW. Bias −0.27 mm, SD 0.93 mm, 95% LOA −2.09 to 1.55 mm. (C) M3 vs M4 for the IVS. Bias −1.07 mm, SD 0.98 mm, 95% LOA −2.99 to 0.84 mm. (D) M3 vs M4 for the LVFW. Bias 0.22 mm, SD 0.96 mm, 95% LOA −1.75 to 2.03 mm. Abbreviations: IVS = interventricular septum; LOA = limits of agreement; LV = left ventricular; LVFW = left ventricular free wall. See Figure 2 for key.

In cats with HCM, thickness of both the IVS and LVFW was different (*P* < .05) among the 4 echocardiographic methods. More specifically, thickness of the IVS was different between M1 and M3 (*P* < .001), M1 and M4 (*P* < .001), M2 and M3 (*P* < .001), and M3 and M4 (*P* < .001). Results of the Bland–Altman analysis comparing subgroups and using 2D vs M-mode images (Figure 4) for IVS revealed a fixed bias of −1.07 to −1.13 mm between methods with 95% LOA between −3.04 and 0.84 mm. Heteroscedasticity (increased disagreement between methods with greater wall thickness) was not observed for all Bland–Altman analyses.

Similar differences (*P* < .05) were found between M1 and M2, M1 and M3, and M1 and M4 for the LVFW in cats with HCM (Table 2). Results of the Bland–Altman analysis comparing subgroups and using 2D vs M-mode images (Figure 4) for LVFW revealed a fixed bias of −0.27 to 0.22 mm between methods with 95% LOA between −2.09 and 2.03 mm in cats with HCM. In addition, and like findings in control cats, the IVS measured consistently thicker than the LVFW for M1 and M3 (*P* < .001) whereas no differences between thickness of the IVS and LVFW were identified using M-mode assessment M2 (*P* = .72) and M4 (*P* = .94).

Results of reliability assessment using intraobserver and interobserver measurement variability analysis for all study variables are summarized in Table 3. Reliability of echocardiographic measurements was excellent with coefficients of variation < 5% (95% CI, 0.9-2.6) for intraobserver measurement variability and excellent or good with coefficients of variation between 2.7% (95% CI, 1.1) and 8.9% (95% CI, 4.3) for interobserver measurement variability.

**Table 3:** Results of the intra and interobserver measurement variability analysis to evaluate test reliability with the CV (mean with 95%CI) and the absolute difference (mean with 95%CI) between repeated measurements in 5 control cats and 5 cats with hypertrophic cardiomyopathy.

Location	Method	Intraobserver		Interobserver	
		CV (%)	Absolute difference (mm)	CV (%)	Absolute difference (mm)
**IVS**	M1	2.7 (.9)	0.17 (0.04)	5.8 (1.4)	0.34 (0.09)
	M2	3.7 (2.6)	0.23 (0.21)	8.9 (4.3)	0.53 (0.21)
	M2	2.2 (1.2)	0.14 (0.06)	5.3 (3.0)	0.33 (0.14)
	M4	3.5 (1.5)	0.20 (0.07)	5.2 (1.9)	0.29 (0.11)
**LVFW**	M1	2.5 (1.2)	0.14 (0.08)	5.9 (2.3)	0.32 (0.11)
	M2	3.2 (1.4)	0.19 (0.08)	7.3 (4.4)	0.42 (0.37)
	M3	4.0 (1.5)	0.21 (0.07)	2.7 (1.1)	0.14 (0.06)
	M4	4.4 (1.8)	0.25 (0.14)	3.7 (2.8)	0.22 (0.18)

Abbreviations: CV = coefficient of variation; IVS = interventricular septum; LVFW = left ventricular free wall.

## Discussion

The main result of our study is that the echocardiographic assessment of LV wall thickness using 2D or M-mode methods and recorded from RPSax or RPLax views lead to statistically significant and clinically relevant differences between recording methods and imaging views and thus cannot be used interchangeably in cats. In addition, thickness of the IVS was larger than the thickness of the LVFW in most cats, either using the 2D or the M-mode technique for image acquisition thus challenging the current use of uniform reference limits independent of acquisition method (<5 mm normal, 5-6 mm equivocal, and > 6 mm abnormal^1^) for septal and free wall thickness in clinical practice.

Reliable and repeatable measurements of LV wall thickness are critical to screening and the diagnosis and follow-up of myocardial disease in cats. Traditionally, measurements of LV wall thickness have been made from 2D-guided M-mode echocardiographic images using the RPSax view.^5^^,^^13^ However, HCM is a disease with substantial morphologic heterogeneity.^2^^,^^6^^,^^14^ Therefore, restricted regional sampling as done with the M-mode technique in a single standard imaging view and only quantifying 2 segments of the LV wall seems ill-defined and prone to error. Other methods such as assessment of LV wall area or LV myocardial volume have rarely been used^2^ but would possibly characterize LV hypertrophy and LV mass more accurately than single 2D wall-thickness estimates. To the authors’ knowledge, validated data on the assessment of LV hypertrophy using echocardiographic variables of myocardial area and volume in cats are currently not available.

While clinicians often have different priorities regarding methods and imaging views to echocardiographically phenotype and quantify the LV in cats, some investigators prefer using 2D images from both the RPLax and RPSax view and selecting the thickest of multiple wall segments to best characterize LV wall dimension in cats. Other investigators solely rely on 2D-guided M-mode measurements. However, data comparing different imaging methods in the echocardiographic evaluation of LV wall thickness in cats are sparse, and it remains largely unknown if different imaging modes and methods can be used interchangeably. The latter would clinically be important not only in the diagnosis of feline cardiomyopathy but also for repeated echocardiographic examinations in the same cat. The 2020 ACVIM consensus statement guidelines for the classification, diagnosis, and management of cardiomyopathies in cats^1^ do not give recommendations as to what methods are preferred in the determination of LV wall thickness. However, the guidelines state that LV wall-thickness measurements using 2D and M-mode techniques are not interchangeable. Similar suggestions have been made elsewhere.^15^^,^^16^ In one study in healthy adult cats,^16^ Bland–Altman analysis revealed only moderate agreement between M-mode and 2D with wide 95% LOA (1.6 mm). In another study in normal cats, differences between 2D and M-mode measurements were up to 1.19 mm.^15^ Limits of agreement between methods in our study were comparatively wide and up to 1.28 mm in healthy control cats but substantially wider (up to 3.05 mm) in cats with HCM.

### Method comparison in control cats

Bias between methods regarding LV wall-thickness measurements in control cats was relatively small (0.04-0.30) with 95% LOA not exceeding 1.28 mm and heteroscedasticity of residuals not identified. Although this difference was statistically significant and above measurement variability (maximum 0.53 mm), at first glance it seems relatively low. However, this bias might change classification of a cat regarding cardiac health status from normal to equivocal or from normal to affected and vice versa. The 2020 ACVIM consensus statement guidelines on cardiomyopathies in cats suggest a LV wall thickness < 5 mm to be considered normal, 5-6 mm equivocal, and > 6 mm abnormal.^1^ That is, the difference between normal and abnormal is just above 1 mm wall-thickness measurement. As an example, using these diagnostic cut-offs, LV wall thickness might turn out unequivocally normal at 4.9 mm using one method but unequivocally abnormal at 6.1 mm using another method.

If myocardial thickness is relatively uniform throughout the LV wall in healthy cats,^17^^,^^18^ there should be reasonable agreement between methods (2D vs M-mode and RPSax vs RPLax). As expected, there was no statistical difference among methods for measurement of the IVS, in contrast to assessment of LVFW thickness. Although bias between methods was small, 95% LOA were relatively wide meaning that in individual cats, the difference between methods can be substantial. Bias was positive for all 4 methods (see Figure 3), indicating that 2D measurements were slightly higher than M-mode measurements in normal cats. Moreover, independent of imaging view, the IVS measured thicker (*P* > .001) than the LVFW in both 2D and M-mode images with a mean difference up to 0.6 mm. This is comparable to findings in previous studies in normal cats where differences between 0.2 and 0.7 mm were reported.^4^^,^^16^^,^^19–23^ Potential reasons include a true difference of myocardial thickness between the IVS and the LVFW as echocardiographically demonstrated in people^24^ and possibly related to the right ventricular contributions in the formation of the IVS,^25^ or systematic measurement error by including 2 endocardial layers for the IVS but only one for the LVFW and right ventricular structures (chordae tendineae, trabecula septomarginalis, and papillary muscles) and LV false tendons in the measurement of the IVS.^9^^,^^23^ Presence of imaging artifacts in the near field might also be considered.^4^^,^^26^

Contrary to IVS thickness, LVFW measurements revealed significantly higher values when assessed by M-mode in comparison to 2D from both the RPLax and RPSax in healthy cats, as reported elsewhere.^22^ To avoid measurement error, we carefully revised the echocardiographic cine loops not to include LV papillary muscles and false tendons when placing the M-mode cursor. However, even if M-mode image acquisition is 2D-guided thus yielding the advantage of excellent temporal resolution due to high frame rate, this modality might overestimate myocardial thickness because of erroneous positioning of the cursor across papillary muscles or including of false tendons^4^^,^^27^ mostly owing to translational motion of the heart relative to a stationary M-mode cursor during recording. The results of the present study suggest not comparing 2D data on LVFW thickness with M-mode data and be consistent using the same echocardiographic method to assess the LVFW during repeated examinations in healthy cats.

### Method comparison in cats with HCM

To the best of our knowledge, comparative studies on different methods of LV wall-thickness measurements in cats with HCM have not been published. We found that 95% LOA between methods were substantially wider in cats with HCM compared to control cats exceeding 3 mm using M1 and 2 mm using M2, M3, and M4. These findings seem clinically relevant as not only diagnosis and classification of heart disease based on LV wall-thickness measurements in cats would be affected, but data on critical change values used for the detection of progression or regression of LV hypertrophy in untreated cats or the effects of medical intervention during longitudinal study would also be misleading. For example, in a recent publication on the effects of the mechanistic target of rapamycin (mTOR) inhibitor rapamycin on LV hypertrophy in cats with HCM,^28^ LV wall thickness was chosen as the therapeutic target. Least squares mean (adjusted average rather than average) maximum wall thickness 6 months after initiation of therapy had increased by 0.44 mm in the placebo group compared to 0.05 mm in the verum group (*P* = .013), and it was concluded that the drug is effective in halting progression of LV hypertrophy in cats with HCM. The study^28^ included repeated echocardiograms, and recording and measurement methods were variable among examinations with both 2D and M-mode as well as IVS and LVFW used interchangeably to determine maximum LV wall thickness. The mean difference of 0.39 mm between the verum and placebo group was small and within the bias and 95% LOA of LV wall-thickness measurements using different methods found in our cohort in cats with HCM. Therefore, it remains unclear as to whether the difference in wall thickness between the verum and placebo groups were induced by the medication or are related to the echocardiographic methods used. This highlights the importance of uniform data acquisition in longitudinal echocardiographic studies.

In cats with HCM, measurements of the IVS using 2D as compared to M-mode yielded significantly higher values with no differences between imaging modes found for the LVFW. While the reason for this finding remains unknown, similar findings have previously been reported.^29^

This study has strengths and limitations. Strengths include the large sample size, the re-evaluation of all echocardiographic measurements with avoidance of using prior data for analysis, consistency of data accrual, completeness of data sets without missing values, and low measurement variability. Limitations include the retrospective nature of the study; possible selection bias due to elimination of poor-quality studies; unrecognized hyperthyroidism, systemic hypertension, and other conditions potentially affecting echocardiographic measurements and group comparisons; unequal matching of groups in particular regarding age and sex; collecting of data initially acquired over a time period of at least 10 years and using different echocardiographic equipment; and potential misclassification of cats owing to the lack of a true gold standard in the diagnosis of HCM. Measurement variability was evaluated from identical cardiac cycles. This might lead to lower variability compared to situations where observers must make random (not identical) selections from multiple stored images. Finally, this study focused on method comparison but cannot give recommendations on which methods are best or preferred for clinical use.

## Conclusions

In conclusion, the main results of this study indicate that different echocardiographic methods in the assessment of LV wall thickness (2D vs M-mode and RPLax vs RPSax imaging views) cannot be used interchangeably, most importantly in cats with HCM where 95% LOA are wide and clinically meaningful. Considering commonly observed heterogeneous distribution of LV hypertrophy, the 2D method might be preferred in the identification of LV wall thickening in cats with HCM. Moreover, in many cats, measurement of thickness of the IVS exceeded measurement of thickness of the LVFW. Our findings underscore the need for collaborative studies to standardize LV wall-thickness measurements in cats, establish generally accepted echocardiographic recording and measurement guidelines, and develop separate decision thresholds for IVS and LVFW thickness.

## Data Availability

The data underlying this article are available in the article and in its supplemental material.

## Off-label antimicrobial declaration

The authors declare no off-label use of antimicrobials.

## Institutional animal care and use committee or other approval declaration

The authors declare no institutional animal care and use committee or other approval was needed.

## Abbreviations

2D: 2-dimensional
95% LOA: 95% limits of agreement
ACVIM: American College of Veterinary Internal Medicine
B-A: Bland–Altman
CV: coefficient of variation
HCM: hypertrophic cardiomyopathy
IVS: interventricular septum
LVFW: left ventricular free wall
RPLax: right parasternal long-axis
RPSax: right parasternal short-axis
